# Socioeconomic deprivation worsens the outcomes of Italian women with hormone receptor-positive breast cancer and decreases the possibility of receiving standard care

**DOI:** 10.18632/oncotarget.19447

**Published:** 2017-07-22

**Authors:** Francesca Di Salvo, Nicola Caranci, Teresa Spadea, Nicolas Zengarini, Pamela Minicozzi, Hade Amash, Mario Fusco, Fabrizio Stracci, Fabio Falcini, Claudia Cirilli, Giuseppina Candela, Rosanna Cusimano, Rosario Tumino, Milena Sant, for the Socioeconomic Inequalities and Oncological Outcomes Italian Working Group

**Affiliations:** ^1^ Analytical Epidemiology and Health Impact Unit, Fondazione IRCCS Istituto Nazionale Tumori, Milan, Italy; ^2^ Agenzia Sanitaria e Sociale Regione Emilia Romagna, Bologna, Italy; ^3^ Servizio Sovrazonale di Epidemiologia ASL Torino 3, Grugliasco, Italy; ^4^ Naples Cancer Registry, ASL Napoli 3 Sud, Brusciano, Italy; ^5^ Umbria Cancer Registry, Public Health Department, University of Perugia, Perugia, Italy; ^6^ Romagna Cancer Registry, IRCCS Istituto Scientifico Romagnolo per lo Studio e la Cura dei Tumori, Meldola, Italy; ^7^ Modena Cancer Registry, Public Health Department AUSL Modena, Modena, Italy; ^8^ Trapani Cancer Registry, Health Prevention Department ASL 9 Trapani, Trapani, Italy; ^9^ Palermo Cancer Registry, Health Science Department University of Palermo, Palermo, Italy; ^10^ Ragusa Cancer Registry, Health Prevention Department ASP Ragusa, Ragusa, Italy

**Keywords:** deprivation index, census tract, sentinel lymph node biopsy, breast-conserving surgery, cancer registry

## Abstract

**Background:**

Socioeconomic factors influence access to cancer care and survival. This study investigated the role of socioeconomic status on the risk of breast cancer recurrence and on the delivery of appropriate cancer care (sentinel lymph node biopsy and breast-conserving surgery plus radiotherapy), by patients’ age and hormone receptor status.

**Methods:**

3,462 breast cancer cases diagnosed in 2003-2005 were selected from 7 Italian cancer registries and assigned to a socioeconomic tertile on the basis of the deprivation index of their census tract. Multivariable models were applied to assess the delivery of sentinel lymph node biopsy and of breast-conserving surgery plus radiotherapy within socioeconomic tertiles.

**Results:**

In the 1,893 women younger than 65 years, the 5-year risk of recurrence was higher in the most deprived group than in the least deprived, but this difference was not significant (16.4% vs. 12.9%, log-rank p=0.08); no difference was seen in women ≥65 years. Among the 2,024 women with hormone receptor-positive cancer, the 5-year risk was significantly higher in the most deprived group than in the least deprived one (13.0% vs. 8.9%, p=0.04); no difference was seen in cases of hormone receptor-negative cancer. The most deprived women were less likely than the least deprived women to receive sentinel lymph node biopsy (adjusted odds ratio (ORa), 0.69; 95% CI, 0.56-0.86) and to undergo breast-conserving surgery plus radiotherapy (ORa=0.66; 95% CI, 0.51-0.86). Conclusions: Socioeconomic inequalities affect the risk of recurrence, among patients with hormone receptor-positive cancer, and the opportunity to receive standard care.

## INTRODUCTION

Breast cancer is the most common cancer in women worldwide. In Europe, breast cancer is estimated to affect more than one in 10 women and accounts for 28.8% of all female cancers [1]. In Italy, it accounts for 29% of newly diagnosed cancers in women, with 48,000 new cases and 12,000 deaths attributed to breast cancer in 2015 [2]. However, there are large variations in access to standard cancer care across Italy, as shown in a population-based study involving 14 cancer registries [3].

Variations in cancer survival have been shown to depend on differences in socioeconomic status (SES), which affects access to cancer screening and high-quality care and, therefore, influences stage at diagnosis and ultimately survival [4, 5]. A recent Italian study [6] found that women with low SES had a significantly lower odds of receiving an annual mammography or clinical breast examination. Another Italian study [7] found that a mammography screening programme, with active invitation of women from the target population, reduced differences in survival. Studies conducted in the United Kingdom found that age at diagnosis of breast cancer influenced the association between SES and survival, as older women with low SES had poorer short-term survival than younger women with high SES [8, 9]. In addition, there is some epidemiological evidence that low SES is associated with breast cancers with an aggressive behaviour [10], and differences in SES may determine differences in exposures to risk factors for different breast cancer subtypes. For instance, women with high SES are more likely to use exogenous hormones and have lower parity [11, 12], two factors associated with the risk of hormone receptor-positive breast cancer. Furthermore, researchers from the United States reported that patients with hormone receptor-negative breast cancer had lower survival rates than patients with hormone receptor-positive subtypes [13], and the associations between SES and both breast cancer incidence [11] and survival [13] vary by tumor subtype, with a significant association for hormone receptor-positive but not for receptor-negative tumors [13].

Only a few studies, all based in the United States, have investigated the influence of census-level SES in predicting breast cancer outcomes by accounting for hormone receptor subtypes and age [11–13]. To further understand how SES impacts upon clinical outcomes, especially in different health care contexts, we used population-based data from Italy to investigate the socioeconomic gradient in the risk of disease recurrence and in the delivery of appropriate breast care according to clinical guidelines, namely sentinel lymph node biopsy and breast-conserving surgery plus radiotherapy [14, 15], taking into account hormone receptor status and age.

## RESULTS

The impact of SES on breast cancer outcomes was assessed in a total of 3,358 Italian women diagnosed in the period 2003-2005 and followed through to 2010 (Table 1). The median age at diagnosis was 62 years and 1,893 women (56.4%) were younger than 65 years. Altogether, 56.3% of cases lived in Southern Italy, and 43.5% had an advanced stage of cancer; 60.3% had a hormone receptor-positive cancer and 16.2% had a hormone receptor-negative cancer. Finally, 74.5% of the sample had a moderate or poor grade of differentiation, and 67.8% received breast-conserving surgery while 31.1% had mastectomy.

**Table 1:** Clinical data for 3,358 Italian women with breast cancer

Variable	Value
Age, median (IQR), years	62 (50-73)
Area of residence, n (%)	
North-Centre	1,466 (43.7)
South	1,892 (56.3)
Stage, n (%)	
Early	1,558 (46.4)
Advanced	1,462 (43.5)
Unknown	338 (10.1)
Hormone receptor status, n (%)	
Positive	2,024 (60.3)
Negative	543 (16.2)
Other or unknown	791 (23.6)
Grade of differentiation, n (%)	
Well	334 (10.0)
Moderate	1,333 (39.7)
Poor	1,170 (34.8)
Unknown	521 (15.5)
Surgery, n (%)	
Breast-conserving surgery	2,277 (67.8)
Mastectomy	1,044 (31.1)
None or unknown	37 (1.1)
Laterality, n (%)	
Right	1,547 (46.1)
Left	1,652 (49.2)
Bilateral	46 (1.4)
Unknown	113 (3.4)

The associations between SES and both area of residence and tumour characteristics were examined separately according to the women's age at diagnosis (Table 2). In women younger than 65 years, residence area, stage at diagnosis and hormone receptor status were not associated with deprivation index (DI). In contrast, women in the most deprived category were more likely than those in the first tertile to have a poorly differentiated cancer (41.2% vs. 33.6%; chi-square p<0.01). Although stage at diagnosis was not found to associate with DI (chi-square, p=0.08), we did observe that women in the most deprived category were more likely than those in the least deprived category to be diagnosed with advanced tumour stage (48.6% vs. 43.5%).

**Table 2:** Characteristics of 3,358 women with breast cancer, by age and deprivation index tertile

Variable	Deprivation index tertile, n (%)^a^			Total	p^b^
	1 (least deprived)	2	3 (most deprived)		
**All patients**	1,304 (38.8)	983 (29.3)	1,071 (31.9)	3,358	
**<65 years (n=1,893)**					
Area					
North-Centre	271 (38.4)	249 (44.4)	262 (41.9)	782 (41.3)	0.09
South	435 (61.6)	312 (55.6)	364 (58.2)	1,111 (58.7)	
Stage					
Early	353 (50.0)	300 (53.5)	286 (45.7)	939 (49.6)	0.08
Advanced	307 (43.5)	233 (41.5)	304 (48.6)	844 (44.6)	
Unknown	46 (6.5)	28 (5.0)	36 (5.8)	110 (5.8)	
Hormone receptor subtype					
Positive	426 (60.4)	333 (59.4)	408 (65.2)	1,167 (61.7)	0.25
Negative	126 (17.9)	108 (19.3)	98 (15.7)	332 (17.5)	
Other or unknown	154 (21.8)	120 (21.4)	120 (19.2)	394 (20.8)	
Grade of differentiation					
Well	72 (10.2)	68 (12.1)	54 (8.6)	194 (10.2)	<0.01
Moderate	285 (40.4)	240 (42.8)	252 (40.3)	777 (41.1)	
Poor	237 (33.6)	191 (34.1)	258 (41.2)	686 (36.3)	
Unknown	112 (15.9)	62 (11.1)	62 (9.9)	236 (12.5)	
Total	706 (37.3)	561 (29.6)	626 (33.1)	1,893 (100)	
**≥65 years (n=1,465)**					
Area					
North-Centre	245 (41.0)	186 (44.1)	253 (56.9)	684 (46.7)	<0.01
South	353 (59.0)	236 (55.9)	192 (43.2)	781 (53.3)	
Stage					
Early	240 (40.1)	177 (41.9)	202 (45.4)	619 (42.3)	0.47
Advanced	261 (43.7)	183 (43.4)	174 (39.1)	618 (42.2)	
Unknown	97 (16.2)	62 (14.7)	69 (15.5)	228 (15.6)	
Hormone receptor subtype					
Positive	337 (56.4)	247 (58.5)	273 (61.4)	857 (58.5)	0.49
Negative	90 (15.1)	57 (13.5)	64 (14.4)	211 (14.4)	
Other or unknown	171 (28.6)	118 (28)	108 (24.3)	397 (27.1)	
Grade of differentiation					
Well	51 (8.5)	39 (9.3)	50 (11.1)	140 (9.5)	0.16
Moderate	221 (37.0)	175 (41.5)	160 (36.0)	556 (38.0)	
Poor	198 (33.1)	126 (29.9)	160 (36.0)	484 (33.0)	
Unknown	128 (21.4)	82 (19.3)	75 (16.9)	285 (19.5)	
Total	598 (40.8)	422 (28.8)	445 (30.4)	1,456 (100)	

^a^Tertiles were calculated at the population level for each region.

^b^Chi-square test.

In women aged ≥65 years, a different pattern emerged (Table 2). Residence area was significantly associated with DI, with 59.0% of least deprived women vs. 43.2% of most deprived women living in the South (chi-square, p<0.01). In contrast, stage, hormone receptor status, and grade did not associate with DI tertiles in older women.

### Outcome 1: risk for recurrence

We assessed the cumulative risk for relapse/distant metastasis in relation to time since breast cancer diagnosis, by age group and hormone receptor status (Figure 2). In women younger than 65 years, the cumulative risk of recurrence was higher in the most deprived group than in the least deprived, but this difference was not significant (16.4% vs. 12.9%, log-rank p=0.08, Figure 2a). In women ≥65 years, there was no relevant difference in cumulative risk at 5 years (13.5% in the most deprived vs. 13.2% in the least deprived groups; p=0.98, Figure 2b). Analysis by hormone receptor status showed that, among women with hormone receptor-positive cancer, the 5-year cumulative risk for recurrence was significantly higher in the most deprived group than in the least deprived one (13.0% vs. 8.9%, p=0.04, Figure 2c), and the medium group overlapped with the most deprived group. In contrast, in hormone receptor-negative breast cancer cases, the 5-year cumulative risk was lower in the most deprived than least deprived women, but this difference was not significant (21.0% vs. 27.5%, p=0.54, Figure 2d). Finally, the 5-year cumulative risk was lower in hormone receptor-positive than in hormone receptor-negative cases in all DI tertiles and altogether (10.7% vs. 24.5%, p<0.01).

**Figure 1:**
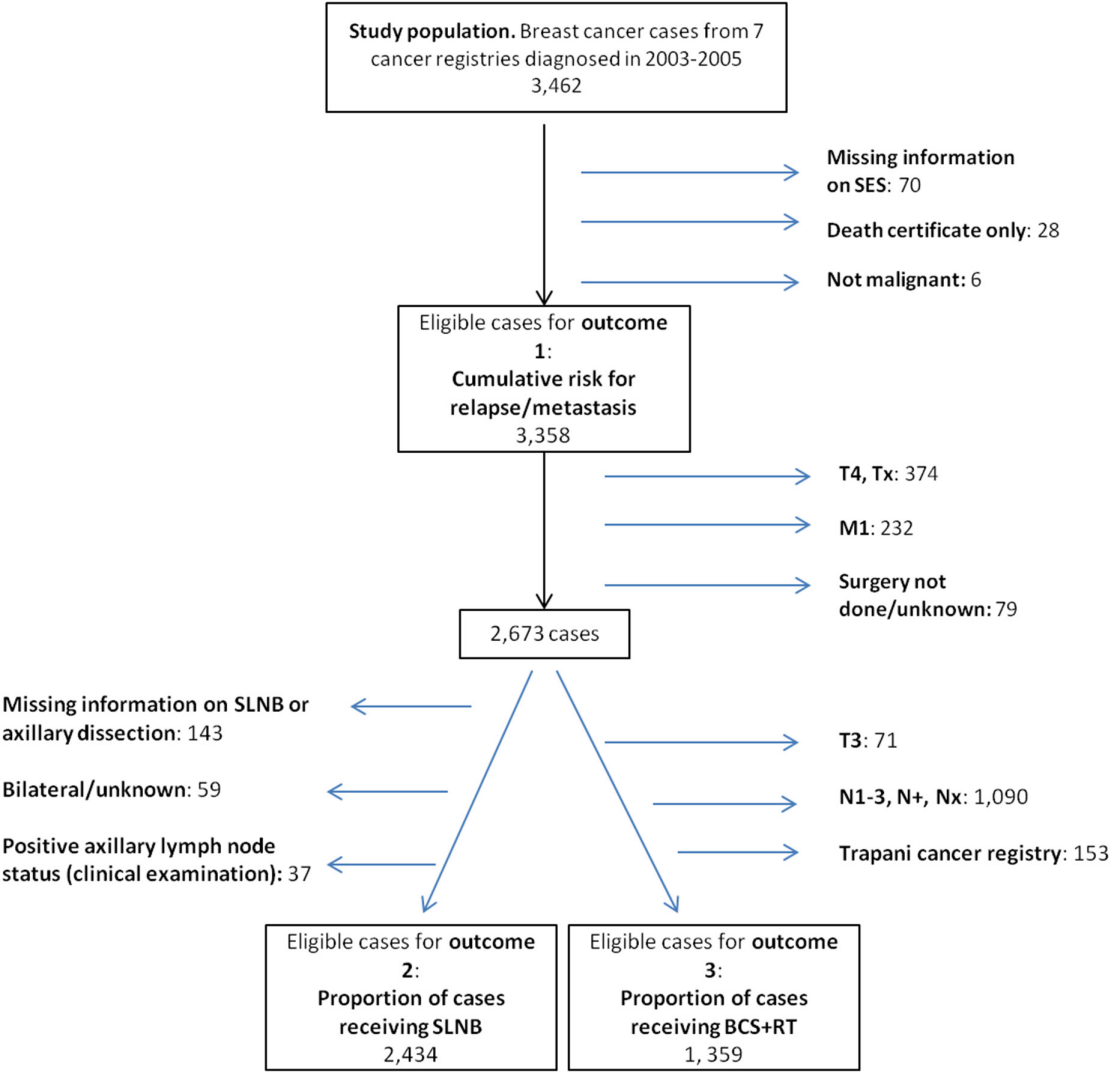
Case selection for three different outcomes SES, socioeconomic status; SLNB, sentinel lymph node biopsy; BCS+RT, breast-conserving surgery plus radiotherapy.

**Figure 2:**
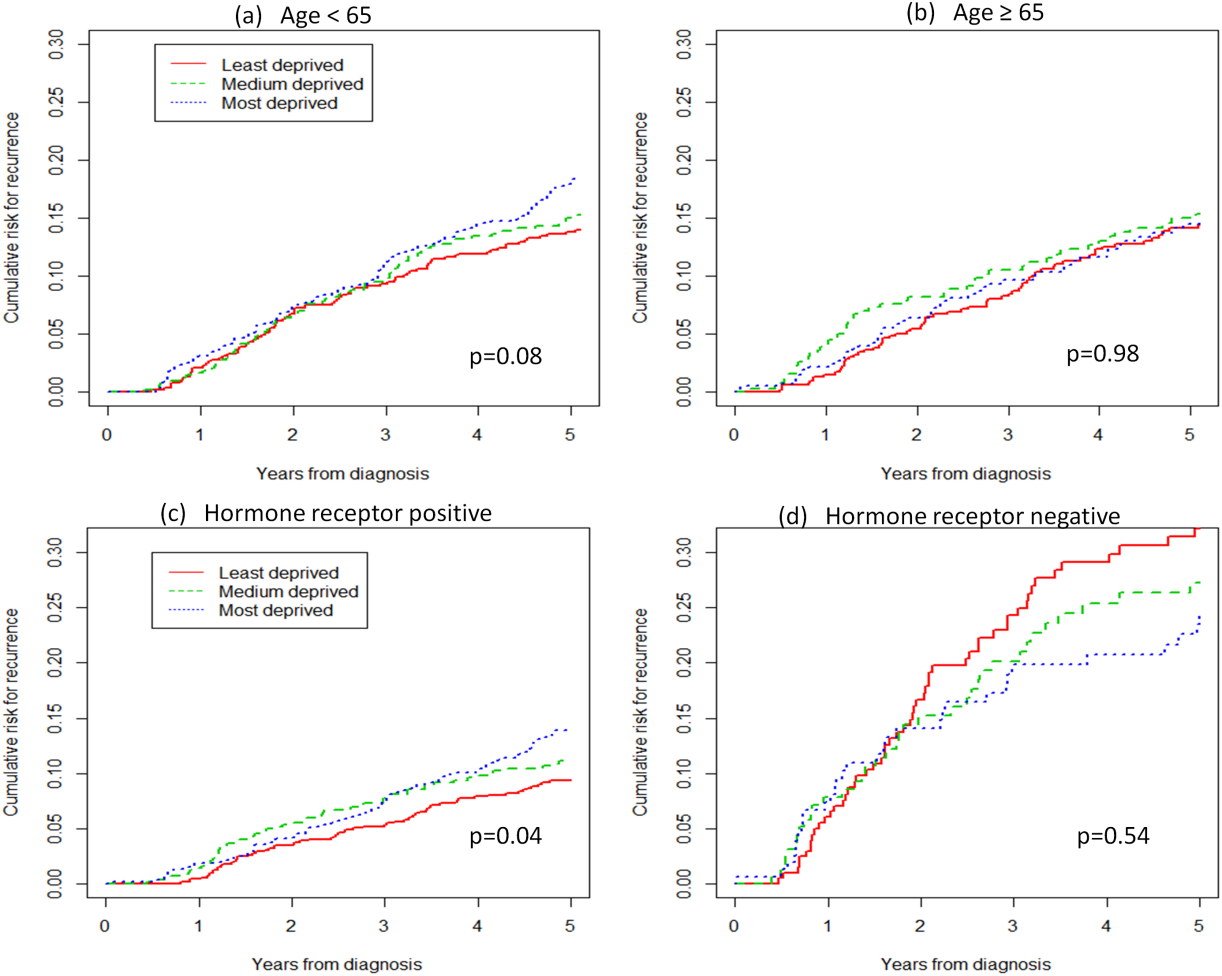
Cumulative risk for locoregional relapse or distant metastasis, by age and hormone receptor status

Table 3 shows the adjusted hazard ratios (HRs) for recurrence according to five Cox regression models. Model 1, which considered all 3,358 cases and adjusts by stage, age, and hormone receptor status, shows that the most deprived women had a non-significant 18% higher risk of recurrence than the least deprived ones (HR=1.18; 95% CI, 0.96-1.46). A late stage at diagnosis carried a significantly higher HR of recurrence than early stage (HR=3.29; 95% CI, 2.69-4.03), and hormone receptor-negative cancers carried a significantly higher risk than hormone receptor-positive subtypes (HR=2.41; 95% CI, 1.95-2.98). Overall, 0.8% of cases were lost of follow-up.

**Table 3:** Adjusted hazard ratios (HRs) for recurrence by age and hormone receptor status, from five Cox regression models

Variable	Age					Hormone receptor				p
	Model 1^a^		Model 2a^b^		Model 2b^c^		Model 3a^d^		Model 3b^e^	
			*<65 years*		*≥65 years*		*Positive*		*Negative*	
	HR (95% CI)	p	HR (95% CI)	p	HR (95% CI)	p	HR (95% CI)	p	HR (95% CI)	
Deprivation index tertile										
1 (least)	1		1		1		1		1	
2	1.07 (0.86-1.34)	0.52	1.13 (0.85-1.50)	0.40	1.00 (0.71-1.42)	0.98	1.18 (0.85-1.63)	0.98	0.83 (0.55-1.26)	0.40
3 (most)	1.18 (0.96-1.46)	0.11	1.29 (0.99-1.68)	0.06	1.04 (0.74-1.46)	0.83	1.44 (1.07-1.93)	0.02	0.74 (0.49-1.14)	0.17
Stage										
Early	1		1		1		1		1	
Late	3.29 (2.69-4.03)	<0.01	3.05 (2.37-3.92)	<0.01	3.72 (2.64-5.24)	<0.01	3.13 (2.36-4.15)	<0.01	3.78 (2.54-5.62)	<0.01
Unknown	1.63 (1.13-2.37)	<0.01	1.77 (1.06-2.98)	0.03	1.57 (0.92-2.70)	0.10	2.17 (1.23-3.81)	<0.01	1.69 (0.71-4.05)	0.24
Age										
<65 years	1		-	-	-	-	1		1	
≥65 years	0.98 (0.81-1.18)	0.86	-	-	-	-	0.95 (0.73-1.23)	0.70	1.03 (0.72-1.48)	0.86
Hormone receptor status										
Positive	1		1		1		-	-	-	-
Negative	2.41 (1.95-2.98)	<0.01	2.31 (1.76-3.02)	<0.01	2.59 (1.82-3.70)	<0.01	-	-	-	-
Other or unknown	1.52 (1.21-1.90)	<0.01	1.48 (1.11-1.97)	<0.01	1.58 (1.11-2.24)	0.01	-	-	-	-

^a^All cases (3,358) were included in this model. ^b^1893 cases aged <65 years were selected for this model adjusted for stage and hormone receptor status. ^c^1465 cases aged ≥65 years were selected for this model adjusted for stage and hormone receptor status. ^d^2,024 hormone receptor-positive cases were selected for this model adjusted for stage and age. ^e^543 hormone receptor-negative cases were selected for this model adjusted for stage and age.

The analysis was repeated with patients stratified by age. In the younger class (Model 2a), SES affected the risk of recurrence, as belonging to the most deprived group carried a 29% higher risk of recurrence after adjusting for stage and hormone receptor status (HR=1.29; 95% CI, 0.99-1.68). In contrast, the risk of recurrence in older women was not affected by SES (Model 2b). For both age classes, a late stage at diagnosis carried a significant higher risk of recurrence than did an early stage: for women <65 years, HR=3.05 (95% CI, 2.37-3.92); for women ≥65 years, HR=3.72 (95% CI, 2.74-5.24). Moreover, a significantly higher risk was found for hormone receptor-negative cancers than hormone receptor-positive ones: for women <65 years, HR=2.31 (95% CI, 1.76-3.02); for women ≥65, HR=2.59 (95% CI, 1.82-3.70).

In a third analysis, patients were stratified according to the hormone receptor status of their cancers. In hormone receptor-positive cases (Model 3a, adjusted by age and stage), the most deprived women had a higher risk of recurrence (HR=1.44; 95% CI, 1.07-1.93) than the least deprived women. Model 3b shows that there was no significant effect of SES on the risk of recurrence in hormone receptor-negative cases (HR=0.74; 95% CI, 0.49-1.14). Late stage posed a significantly higher risk of recurrence for both hormone receptor-positive women (HR=3.13; 95% CI, 2.36-4.15) and hormone receptor-negative women (HR=3.78; 95% CI, 2.54-5.62). No differences were seen by age.

In order to investigate whether socioeconomic deprivation influenced the outcome of women eligible to participate in mass screening programmes, we also estimated HRs for three age classes: <50 years, 50-69 years, and ≥70 years; the central class represents the target population of the screening programme in Italy. We found a significant association between advanced stage at diagnosis and SES in the youngest age class (Supplementary Table 1), and a significant association between grade and SES in the central age class (Supplementary Table 2). No association between SES and stage or grade was found in the oldest age group, a significant association was found with the residence area (Supplementary Table 3). However, in multivariable regression models adjusted for stage and hormone receptor status, no significant effect of SES on the risk of recurrence was found in any age group (Supplementary Table 4).

### Outcomes 2 and 3: appropriateness of care

To investigate the relationship between SES and the probability of receiving sentinel lymph node biopsy (SLNB, Outcome 2), we analysed 2,434 cases, selected on the basis of clinical characteristics (Figure 1), for whom this procedure is recommended. Overall, 810 women (33.3%) received SLNB. The percentages of women who underwent SLNB were 29.8% in the most deprived group and 34.8% in the least deprived one (Table 4). The odds ratio (ORa) for receiving SLNB, adjusted by residence area, age, and hormone receptor status, was significantly lower in the most deprived women than in the least deprived (ORa=0.69; 95% CI, 0.56-0.86). SLBN was less commonly done in women resident in the South vs. those living in the North-Centre (ORa=0.27; 95% CI, 0.22-0.32), in older than younger women (ORa=0.57; 95% CI, 0.47-0.69), and in women with hormone receptor-negative cancer (ORa=0.51; 95% CI, 0.40-0.67).

**Table 4:** Cases receiving appropriate care and results of multivariable logistic regression

	SLNB (n = 2,434)			BCS+RT (n = 1,359)		
	n (%)	ORa (95% CI)	p	n (%)	ORa (95% CI)	p
Deprivation index tertile						
1 (least)	318 (34.8)	1		326 (65.3)	1	
2	256 (35.2)	0.93 (0.75-1.16)	0.53	277 (69.4)	1.17 (0.88-1.56)	0.29
3 (most)	236 (29.8)	0.69 (0.56-0.86)	<0.01	258 (56.0)	0.66 (0.51-0.86)	<0.01
Area						
North-Centre	542 (47.4)	1		485 (63.7)	1	
South	268 (20.8)	0.27 (0.22-0.32)	<0.01	376 (63.0)	0.93 (0.73-1.17)	0.52
Age						
<65 years	446 (36.6)	1		466 (72.0)	1	
≥65 years	364 (29.9)	0.57 (0.47-0.69)	<0.01	395 (55.5)	0.45 (0.36-0.57)	<0.01
Hormone receptor status						
Positive	573 (35.7)	1		594 (64.9)	1	
Negative	96 (23.2)	0.51 (0.40-0.67)	<0.01	136 (61.5)	0.80 (0.59-1.10)	0.17
Other or unknown	141 (34.1)	0.80 (0.63-1.02)	0.08	131 (59.0)	0.75 (0.55-1.02)	0.07

ORa, adjusted odds ratio; CI, confidence interval; SLNB, sentinel lymph node biopsy; BCS+RT, breast-conserving surgery plus radiotherapy.

To investigate the relationship between SES and the chance to receive breast-conserving surgery plus radiotherapy (BCS+RT, Outcome 3), we analysed 1,359 breast cancer cases (Table 4) selected on the basis of clinical characteristics (Figure 1), for whom this procedure is recommended. Overall, 861 women (63.4%) received this treatment. The percentages of women who received BCS+RT were 65.3% in the least deprived group and 56.0% in most deprived group, with higher rates in the central group. The ORa of receiving BCS+RT, adjusted by area, age and hormone receptor status, was significantly lower in the most deprived women than in the least deprived (ORa=0.66; 95% CI, 0.51-0.86). Furthermore, older women were less likely to receive BCS+RT than younger women (ORa=0.45; 95% CI, 0.36-0.57). Living in the South of Italy and having a hormone receptor-negative cancer subtype did not affect the opportunity to receive BCS+RT.

## DISCUSSION

This study examined the impact of SES, scored with a deprivation index, on three breast cancer outcomes in Italy. Analysing the first outcome, we found that, for all cases, the most deprived women had a non-significant 18% higher risk of recurrence than the least deprived ones; however, within the group of hormone receptor-positive cancer, the most deprived ones had a significantly higher risk of recurrence than the least deprived. Also, for women younger than 65 years, the most deprived had a significantly higher frequency of poorly differentiated breast cancer at diagnosis and, consequently, a higher risk of recurrence than the least deprived cases. Investigating Outcomes 2 and 3, we observed that women in the most deprived tertile received SNLB and BCS+RT less frequently than the least deprived women.

Italy has a public national health care system, financed by general taxation, which should “guarantee the uniform provision of comprehensive care and assistance throughout the country” [16]. However, there are differences in the organisation and provision of health care among the 20 regions, because the central government defines the “essential levels of care” that are guaranteed to all residents, while the regions, which differ in economic resources, specialised hospitals and health expenditures, are responsible for administering the publicly financed care. SLNB and BCS+RT should be performed regardless of a patient's ability to pay. In reality, however, they are provided mostly by specialised cancer centres (which tend to be found in wealthier areas) rather than in low-volume hospitals. Thus, although delivery of these forms of care should not be influenced by SES, this study showed that the most deprived women were less likely to undergo these procedures. One explanation for this difference is the out-of-pocket costs that cancer survivors must bear in order to reach specialised cancer hospitals [17]. Another explanation is that, in this study, the DI was assigned to cases according to their area of residence; thus a patient was defined as “deprived” because she lives in a deprived area. As a result, more advantaged patients, who have relatively little trouble affording these expenses and who may live closer to specialised centres, have more chances to receive standard care.

Studies in the United States [18] and Italy [19] found that advanced age was associated with getting less care according to treatment guidelines, irrespective of comorbidities, stage, or tumour characteristics. Moreover, in the United States, women aged 65 years or more received radiation therapy less frequently than younger women [18]. Our results are consistent with these studies, in that we also found that elderly patients were treated less frequently with BCS+RT than younger women. Furthermore, we observed that, within a single age group, socioeconomic deprivation independently affected the possibility of receiving this standard treatment.

This study found that SLNB was performed less frequently in older than younger patients and in the most deprived than the least deprived. Radhakrishnan et al. [20] reported that, in the United States, old age was associated with a decreased odds of receiving SLNB for early breast cancer. This undertreatment does not necessarily result in worse outcomes. In fact, some studies found no effect on disease-free survival in elderly patients in whom lymph node evaluation was omitted [21–23].

Our study revealed that SES affects the risk of disease recurrence differentially according to breast cancer subtype, with a significant difference only in the hormone receptor-positive group. In the reverse Kaplan-Meier analysis for cumulative probability (Figure 2c), the curve for least deprived women is always below that for the most deprived women, which overlaps and crosses the curve for the medium deprived women. This effect may be due to the fact that calculation of the medium curve is more influenced by neighboring values than the two others. Furthermore, these curves derive from univariate analyses, not adjusted for any prognostic factors. When we estimated Cox models adjusting for stage at diagnosis and age, we found that the most deprived women had a significantly higher risk than the least deprived in the subgroup of hormone receptor-positive cancer but not in that of hormone receptor-negative cancer. We do not have an explanation for this phenomenon, and the relatively low number of women with hormone receptor-negative breast cancer prevents definitive conclusions. However, this difference may be explained by the fact that the latter subtype (which includes triple-negative breast cancer and a large part of HER2-positive breast cancer) is intrinsically more aggressive, less respondent to therapies, and likely diagnosed at a more advanced stage than hormone receptor-positive cancers [24]. Therefore, hormone receptor-negative tumours carry a high recurrence risk, regardless of SES.

This finding is consistent with another study by Akinyemiju et al. [13], reporting that the higher mortality risk in hormone receptor-negative cancers did not change after adjustment for SES.

Furthermore, a US study [25] found that survival differences by ethnicity are more marked for less aggressive cancers than more aggressive ones. Thus, black women in the United States had lower survival than whites for most breast cancer subtypes, but among the aggressive triple-negative subtype, black women had similar survival to women of other races, suggesting that this subtype of breast cancer has worse prognosis regardless of other factors as ethinicity.

In our study, we did not find a significant association between SES and hormone receptor subtype. In contrast, Andaya et al. [26] found that, in the United States, low SES areas tended to have a higher prevalence of cancers with hormone receptor-negative status than did the more affluent areas. We found that, in the <65 years age group, women in the most deprived category were more likely than the least deprived to be diagnosed with advanced tumour stage and with a poorly differentiated cancer and had a non-significant increased risk of recurrence. The association between socioeconomic deprivation and advanced stage may depend on a diagnostic delay due to scarce access to diagnostic facilities, while wealthier persons may have easier access to diagnostic facilities, health information and medical examinations.

Several European studies found that a population-based breast screening programme had a significant impact on the socioeconomic gradient in survival [27–29]. In Italy, screening programmes covered approximately 75% of the female population aged 50-69 years in 2005 [30]. After implementation of the screening programme in Emilia-Romagna Region, the survival disadvantage of low educated patients (compared with highly educated patients) disappeared among women in the age group invited to screening [7]. Similar results were found by a study conducted in the City of Florence [31]. Furthermore, in the City of Turin, breast cancer patients diagnosed in a screening programme received good-quality breast cancer care without differences by SES [32].

We did not find a significant association between SES and the risk of disease recurrence in the group of women aged less than 50 years or in the other two age groups. We found that women with low SES were more likely to have a more advanced tumour stage only in the youngest age group, so we can assume that the effect of SES on tumour stage may be attenuated for women aged between 50 and 69 years old, who represent the target of the screening programme. A Scottish study [8] that investigated the effect of SES on breast cancer survival found survival differences of 10% between rich and deprived women, but there was no evidence that the effect of deprivation on survival varied significantly by age group. A more recent study in England [9] reported that the socioeconomic deprivation gap in survival widened with increasing age at diagnosis.

### Strengths and weakness

Our analysis was strengthened by the use of the population-based EUROCARE 5-High Resolution database, which provides a representative sample of all breast cancer cases incident in the registry areas. This feature allowed our study to avoid selection bias due to recruitment in a single institution. Furthermore, for the first time in Italy, we linked population-based data with information on care and treatment to the census tract DI database [33], in order to attribute to each cancer case a SES category. A limitation of our study regards the use of a census tract DI rather than an individual DI because, as argued by Moriceau et al. [34], the association between SES and cancer survival, where it exists, was stronger when SES was assessed individually [35] than according to the census tracts [36]. Indeed, people can live in a deprived area without being deprived themselves. Another limitation of this study is that we did not have information on comorbidities, screening, or lifestyle and environmental factors that interact with SES and potentially affect prognosis and therefore survival.

We decided to categorise breast cancer subtypes into two main groups, hormone receptor positive and negative, instead of using a more detailed classification, e.g. distinguishing luminal types, and incorporating HER2 and ki67. We used this broad classification to have an adequate number of cases in each analysed grouping, to reduce variability due to low numbers.

In summary, our study confirms the existence of health inequalities across Italy and suggests that those inequalities affect breast cancer outcomes, such as risk of recurrence and opportunity to receive standard care. Through the use of a census tract DI, this study identified, within small geographic areas, groups of people to whom actions to reduce socioeconomic inequalities should be directed, in order to extend the availability of appropriate care and correct management of breast cancer patients.

## MATERIALS AND METHODS

### Study design and data sources

This study was conducted in the context of EUROCARE (European Cancer Registry-Based Study on Survival and Care of Cancer Patients) [37] and was based on data already collected for a EUROCARE-5 High Resolution Study of breast cancer survival in Italy [38]. According to the EUROCARE-High Resolution study protocol [39], a cancer registry may participate if it is able to provide data for at least 500 cases of primary breast cancer, diagnosed in women (≥15 years old) in the period 2003–2005 and followed to the end of 2010. Of the 34 cancer registries belonging to the Italian Cancer Registry Association (AIRTum) [40], nine contributed data for the High Resolution study and seven of these nine cancer registries also participated in the present study. Three of the participating cancer registries cover areas of North-Central Italy: Modena Cancer Registry and Romagna Cancer Registry (Region of Emilia-Romagna), and Umbria Cancer Registry (Umbria). The other four registries cover Southern Italy: Napoli Cancer Registry (Campania) and the registries of Palermo, Ragusa, Trapani (Sicily).

Cases were extracted from the AIRTum database by a randomised procedure balanced for each participating registry and year of diagnosis. From each participating cancer registry, we obtained clinical and socioeconomic data for about 500 cases, according to the EUROCARE-5 High Resolution Study protocol [39], for a total of 3462 cases. For the present study, cancer registries were asked to provide the 2001 census tract [41] of residence for each case.

### Clinical data

For each case included in the study, we obtained information on age and stage at diagnosis, clinical and pathological characteristics of the tumour, treatments given (breast-conserving surgery, mastectomy, radiotherapy), and data on disease progression and life status (alive or dead) up to 5 years after the diagnosis.

Age at diagnosis was dichotomised into two classes (<65 years, ≥65 years) to compare young and elderly people, while three age classes (<50 years, 50–69 years, ≥70 years) were used to examine the impact of breast cancer screening programmes available for women in the 50–69 year group [30]. Stage at diagnosis was coded according to the TNM system (sixth edition) [42] and categorised as early stage (T1N0M0 or T2-3N0M0), advanced stage (T1-3N+M0 or T4anyNM0-1), or unknown. Tumour grade was classified as well differentiated, moderately differentiated, poorly differentiated, or unknown. Tumour laterality was classified as right, left, bilateral, or unknown.

Tumours were scored positive for estrogen or progesterone receptor expression when 10% or more of neoplastic cells had nuclear immunohistochemical staining. Hormone receptor status was then considered hormone receptor positive when the tumour was positive for both estrogen receptor and progesterone receptor, hormone receptor negative when staining was negative for both receptors, and other in the remaining cases; use of this broad classification was motivated by the need to have an adequate number of cases in each group.

### Socioeconomic status data

The SES of each patient was expressed using the Italian deprivation index (DI), a score recently developed by some of us [33]. This index is a sum of the frequencies of five census variables that reflect social and material deprivation: low level of education, unemployment, one-parent family, home rental and home overcrowding. Current values of the index are based on data from the 2001 Italian population census and refer to 352,605 census tracts (average of 169 inhabitants in a mean area of 0.6 km^2^). Because the Italian DI at census-tract level was shown to represent individual deprivation [43], it was used as the socioeconomic indicator for individual patients in this study. The census tract in which each patient lived at the time of diagnosis was provided by participating cancer registries, and the corresponding DI was obtained from the database held by Caranci et al. [33].

To study the effects of socioeconomic status, we divided the population in tertiles, a classification system that provides clear contrast between groups [32]. To this aim, we first determined cut-off values of DI for tertiles in each of the four Italian regions considered in the study. Then, patients were assigned to one of these tertiles on the basis of the DI for their census tract. The first tertile included the least deprived cases and the third tertile had the most deprived cases.

### Outcome variables and case selection

In order to investigate the influence of SES on prognosis and adhesion to selected clinical recommendations, we considered three primary outcomes: 1) Cumulative risk of recurrence, by age and hormone receptor status; 2) Proportion of cases that underwent sentinel lymph node biopsy (SLNB); and 3) Proportion of T1-2N0M0 cases that underwent breast-conserving surgery plus radiotherapy (BCS+RT).

To study these outcome variables, a series of increasingly stringent exclusion criteria were applied to select cases from the initial study population of 3,462 cases (Figure 1). First, we excluded 70 cases for whom data on SES were missing, 28 cases who were known to the registry only because breast cancer had been mentioned on their death certificates, and 6 cases with benign breast disease. Therefore, 3,358 women were analysed for Outcome 1 (risk of recurrence).

To study Outcomes 2 and 3, we further excluded 374 cases with stage T4 or unknown T stage (Tx), 232 cases with metastatic disease at diagnosis (M1), and 79 cases that were not operated. These exclusion criteria left a set of 2,673 cases for analysis of the appropriateness of care (Figure 1). To study Outcome 2 (proportion of cases receiving SLNB), we excluded 143 cases for whom information on SLNB or axillary dissection was incomplete, 59 cases with bilateral breast cancer or unknown laterality (this decision was taken because we did not find strong recommendations regarding delivery of SLNB to bilateral breast cancer), and 37 cases in whom clinical examination revealed positive axillary lymph node status, in accordance with clinical guidelines; thus 2,434 selected cases were assessed for Outcome 2. Finally, for Outcome 3 (proportion of cases receiving BCS+RT), we excluded cases with stage T3, nodal metastases at diagnosis (in accordance with ESMO guidelines [15]), and all cases from the Trapani Cancer Registry because more than 50% had missing or incomplete information on radiotherapy. After these exclusions, 1,359 cases with T1-2N0M0 disease were available for analysis. The groups assessed for Outcomes 1, 2 and 3 were similar in baseline characteristics.

### Statistical analysis

Descriptive statistics were calculated to summarise demographic and prognostic factors of patients. The associations between DI tertiles and both residence area and various tumour characteristics were assessed using the chi-square test. These analyses were done separately for patients <65 years and those ≥65 years of age at diagnosis. Tests were two-sided and a difference with a p<0.05 was considered statistically significant.

The cumulative risks of recurrence in the first 5 years after diagnosis, for different DI tertiles, were estimated using a reverse Kaplan-Meier method for cumulative probability. Every locoregional relapse or distant metastasis was considered an event, and time between diagnosis and event was considered as survival time. Log-rank tests were used to evaluate differences by DI tertiles. Cox proportional hazard models were applied to study the impact of SES on the risk of developing progression of disease. The impact of prognostic factors was estimated by subsequently adding in the models age, stage and hormone receptor status. We stratified by hormone receptor status because one study found that hormone receptor-negative cancers were more frequent in deprived areas and carried a poor prognosis independent of SES [11]. We stratified by age because, in younger women, the distribution of cases by SES and prognostic factors (stage and grade) showed a higher percentage of cases with advanced stage (48.6% vs. 43.5%) and poorly differentiated grade (33.6% vs. 42.2%) in the most deprived class than in the least deprived. Therefore the relation between SES and disease recurrence may be influenced by age. Multivariable logistic regression models were used to investigate the association between DI tertiles and the delivery of appropriate care. In particular, we assessed the delivery of SLNB and BCS+RT to the cases assessed for Outcomes 2 and 3, respectively, controlling for age, hormone receptor status, and residence area. We included in the model those variables that had a significant impact on outcome in the univariate analysis and that are known risk factors for that outcome. Statistical analyses were carried out using the Stata statistical package (version 12) and R (version 3.3.1).

## SUPPLEMENTARY TABLES



## Abbreviations

BCS+RT: breast-conserving surgery plus radiotherapy
DI: deprivation index
SES: socioeconomic status
SLNB: sentinel lymph node biopsy
