# Supplementary material for: Socioeconomic deprivation worsens the outcomes of Italian women with hormone receptor-positive breast cancer and decreases the possibility of receiving standard care
Source: Oncotarget. 2017 Jul 22;8(40):68402–14. doi: 10.18632/oncotarget.19447 (PMC5620266; doi:10.18632/oncotarget.19447)
Supplement: Supplementary file 1 [file oncotarget-08-68402-s001.pdf]

Socioeconomic deprivation worsens the outcomes of Italian women with hormone receptor-positive breast cancer and decreases the possibility of receiving standard care

SUPPLEMENTARY MATERIALS

Supplementary Table 1: Characteristics of 794 patients aged <50 years, by deprivation index[a]

	Deprivation index tertile				
	1 (least dep)	***2***	***3 (most dep)***	**Total**	**p[b]**
Area					
Centre-North	98 (35.3)	92 (40.5)	116 (40.1)	306 (38.5)	0.38
South	180 (64.8)	135 (59.5)	173 (59.9)	488 (61.5)	
Stage					
Early	146 (52.5)	118 (52)	120 (41.5)	384 (48.4)	0.02
Advanced	114 (41)	102 (44.9)	154 (53.3)	370 (46.6)	
unknown	18 (6.5)	7 (3.1)	15 (5.2)	40 (5)	
Hormone receptor status					
positive	172 (61.9)	148 (65.2)	190 (65.7)	510 (64.2)	0.58
negative	49 (17.6)	44 (19.4)	47 (16.3)	140 (17.6)	
Other/unknown	57 (20.5)	35 (15.4)	52 (18)	144 (18.1)	
Grading					
Well different	26 (9.4)	26 (11.5)	25 (8.7)	77 (9.7)	0.32
Moderately different	109 (39.2)	94 (41.4)	115 (39.8)	318 (40.1)	
Poorly differentiated	100 (36)	81 (35.7)	121 (41.9)	302 (38)	
unknown	43 (15.5)	26 (11.5)	28 (9.7)	97 (12.2)	
Total	228 (35)	227 (28.6)	289 (36.4)	794 (100)	

[a]Classes were calculated by tertiles of resident population (at regional level, in 2001): 1=least deprived, 2=medium, 3=most deprived.

[b]Chi-square test for categorical variables.

Supplementary Table 2: Characteristics of 1,472 patients aged 50-70 years, by deprivation index[a]

	Deprivation index tertile				
	1 (least dep)	*2*	*3 (most dep)*	**Total**	**p[b]**
Area					
Centre-North	231 (40.4)	205 (46.1)	203 (44.6)	639 (43.4)	0.16
South	341 (59.6)	240 (53.9)	252 (55.4)	833 (56.6)	
Stage					
Early	284 (49.6)	250 (56.2)	228 (50.1)	762 (51.8)	0.27
Advanced	249 (43.5)	171 (38.4)	198 (43.5)	618 (42.0)	
unknown	39 (6.9)	24 (5.4)	29 (6.4)	92 (6.2)	
Hormone receptor status					
positive	337 (58.9)	259 (58.2)	296 (65.1)	892 (60.6)	0.23
negative	98 (17.1)	77 (17.3)	67 (14.7)	242 (16.4)	
Other/unknown	137 (24.0)	109 (24.5)	92 (20.2)	338 (23.0)	
Grading					
Well different	58 (10.1)	54 (12.1)	48 (10.6)	160 (10.9)	0.02
Moderately different	241 (42.1)	205 (46.1)	176 (38.7)	622 (42.3)	
Poorly differentiated	189 (33.1)	135 (30.3)	184 (40.4)	508 (34.5)	
unknown	84 (14.7)	51 (11.5)	47 (10.3)	182 (12.4)	
Total	572 (38.9)	445 (30.3)	455 (30.9)	1,472 (100)	

[a]Classes were calculated by tertiles of resident population (at regional level, in 2001): 1=least deprived, 2=medium, 3=most deprived.

[b]Chi-square test for categorical variables.

Supplementary Table 3: Characteristics of 1,092 patients aged more than 70 year, by deprivation index[a]

	Deprivation index tertile				
	1 (least dep)	***2***	***3 (most dep)***	**Total**	**p[b]**
Area					
Centre-North	187 (41.2)	138 (44.4)	196 (60)	521 (47.7)	<0.01
South	267 (58.8)	173 (55.6)	131 (40)	571 (52.3)	
Stage					
Early	163 (35.9)	109 (35.1)	140 (42.8)	142 (37.7)	
Advanced	205 (45.2)	143 (46.0)	126 (38.5)	474 (43.4)	0.21
unknown	86 (18.9)	59 (18.9)	61 (18.7)	206 (18.9)	
Hormone receptor status					
positive	254 (56.0)	173 (55.6)	195 (59.6)	622 (57.0)	
negative	69 (15.2)	44 (14.2)	48 (14.7)	161 (14.7)	0.74
Other/unknown	131 (28.8)	94 (30.2)	84 (25.7)	309 (28.3)	
Grading					
Well different	39 (8.9)	27 (8.7)	31 (9.5)	97 (8.9)	
Moderately different	156 (34.4)	116 (37.3)	121 (37.0)	393 (36.0)	0.65
Poorly differentiated	146 (32.2)	101 (32.5)	113 (34.6)	360 (32.9)	
unknown	113 (24.9)	67 (21.5)	62 (18.9)	242 (22.2)	
Total	454 (41.5)	311 (28.5)	327 (30.0)	1,092 (100)	

[a]Classes were calculated by tertiles of resident population (at regional level, in 2001): 1=least deprived, 2=medium, 3=most deprived.
[b]Chi-square test for categorical variables.

Supplementary Table 4: Adjusted hazard ratios (HR) for relapse or metastasis, by age class

	Age					
	Model 2a[a]		Model 2b[b]		Model 2c[c]	
	<50 years		*50-70 years*		*70+ years*	
	**HR**	**95% CI**	**HR**	**95% CI**	**HR**	**95% CI**
Deprivation index tertile						
1 (least dep)	1		1		1	
2	0.98	0.61-1.58	1.11	0.81-1.53	1.11	0.75-1.64
3 (most dep)	1.26	0.82-1.91	1.18	0.87-1.61	1.16	0.78-1.71
Stage						
Early	1		1		1	
Late	2.74	1.84-4.09	3.57	2.66-4.77	3.36	2.25-5.01
unknown	1.21	0.47-3.14	1.91	1.04-3.50	1.55	0.88-2.73
Hormone receptor status						
positive	1		1		1	
negative	1.81	1.16-2.83	2.62	1.92-3.56	2.69	1.81-4.01
Other/ unknown	1.60	1.03-2.51	1.38	0.99-1.91	1.66	1.11-2.47

[a]794 cases aged <50 years were selected for this model adjusted for stage and hormone-receptor subtype. [b]1472 cases aged 50-70 years were selected for this model adjusted for stage and hormone-receptor subtype. [c]1,092 cases aged 70+ years were selected for this model adjusted for stage and hormone-receptor subtype.
